# The insider's perspective: The intracellular complosome and immune cell dynamics in cancer

**DOI:** 10.1002/ctm2.70628

**Published:** 2026-02-20

**Authors:** Alexandra Bennion, Joanne Lysaght, Niamh Lynam‐Lennon

**Affiliations:** ^1^ Department of Surgery School of Medicine Trinity St. James's Cancer Institute and Trinity Translational Medicine Institute (TTMI) St. James's Hospital Trinity College Dublin Dublin Ireland; ^2^ Cancer Immunology and Immunotherapy Group School of Medicine Trinity St. James's Cancer Institute and Trinity Translational Medicine Institute (TTMI) St. James's Hospital Trinity College Dublin Dublin Ireland; ^3^ Department of Biology Maynooth University Maynooth Ireland; ^4^ Kathleen Lonsdale Institute for Human Health Research Maynooth University Maynooth Ireland

**Keywords:** C3, C3a, C5, C5a, cancer immunity, complement system, complosome, immune cells, immunotherapy, intracellular complement, tumour microenvironment

## Abstract

Complement is increasingly recognised as a driver and modulator of antitumour immunity, with context‐dependent effects across T cells, myeloid subsets, stromal elements and tumour cells. Although best known for pathogen clearance and membrane attack complex (MAC) formation, complement also acts intracellularly via the ‘complosome’ to regulate cellular homeostasis and gene expression. Complosome activity may dampen antitumour responses by rewiring single‐cell metabolism and transcription, altering nutrient flux and fostering an immunosuppressive microenvironment. Here, we synthesise advances in intracellular and extracellular complement, with emphasis on complement component 3 (C3) and receptors (C3aR1, C5aR1/CD88, C5aR2/C5L2), highlighting how these pathways shape T‐cell metabolism, exhaustion programmes and inflammatory tone within tumours. Evidence indicates that tonic C3/C5 signalling restrains cytotoxicity via C5aR1‐driven myeloid recruitment and cytokine cascades, while complosome signalling tunes T‐cell activation thresholds and bioenergetics. We outline considerations for selectively modulating intracellular versus extracellular complement, propose cell‐type‐resolved biomarker strategies and identify opportunities for complosome‐directed therapies in cancer, integrating roles across T cells, macrophages, B cells, neutrophils, NK cells, regulatory T cells, dendritic cells, myeloid‐derived suppressor cells and cancer‐associated fibroblasts.

**Key points:**

- Intracellular complement (complosome) shapes the tumor immune microenvironment.
- Complosome's role in cancer is underrecognized yet central to tumor immunity.
- C3/C5‐driven complosome signals rewire T cell activation, fate, and metabolism. Complosome activity can promote pro‐tumor immune cell function.
- Blocking the complosome, alone or with checkpoint inhibitors, unveils a new tumor target.

## Graphical abstract

Intracellular complement (complosome) shapes the tumour immune microenvironment.

Complosome's role in cancer is underrecognised yet central to tumour immunity.

Complosome activity can promote pro‐tumour immune cell function.

Blocking the complosome, alone or with checkpoint inhibitors, unveils a new tumour target.

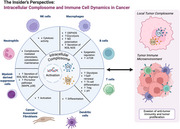

## 1 INTRODUCTION TO THE COMPLEMENT SYSTEM

One of the oldest and most important components of innate immunity is the activation of the complement system, a cluster of over 50 highly conserved, membrane‐bound and intracellular proteins that primarily circulate in blood and lymph, opsonise pathogens and induce inflammatory responses.^1^ Initially described as a ‘complement’ to enhance and support antibodies in the detection and removal of pathogens, it is now understood that the complement system can independently trigger immunosurveillance.

**FIGURE 1:**
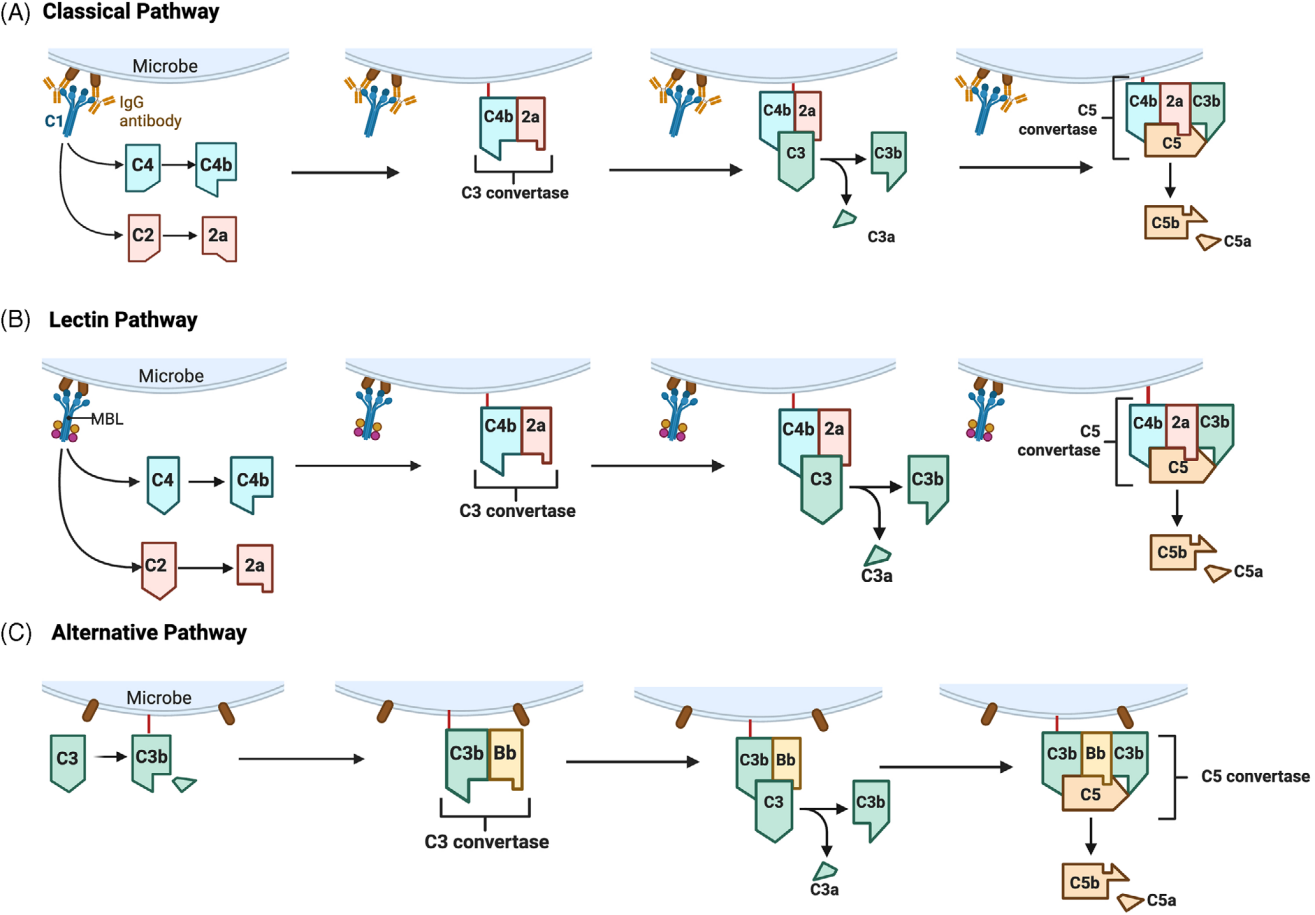
Overview of the three main complement activation pathways including the (A) classical pathway, (B) lectin pathway and (C) alternative pathway. All three pathways converge at the cleavage of C3. Unlike the classical or lectin pathway, the alternative pathway is continuously active at low levels due to spontaneous hydrolysis of C3 thioester and utilises a distinct C3 convertase comprised of C3b and a cleaved fragment of plasma protein Factor B. When C3b is bound to a pathogen surface, Factor B binds and is cleaved by plasma protein Factor D, resulting in C3bBb (C3 convertase). Complement may also be activated via the coagulation cascade pathway (tissue damage) Factor XII_a,_ which upregulates C1 in the classical pathway, or via inducible serine proteases and proteins (thrombin, plasmin) activated by macrophages.^148^ Created with BioRender.com.

Three known canonical pathways activate liver‐derived and serum‐effective complement in the extracellular space: (i) the classical (antibody‐dependent) pathway, (ii) the lectin (carbohydrate recognition) pathway, and (iii) the alternative pathway activated continuously at low levels^2^ (Figure 1).

**FIGURE 2:**
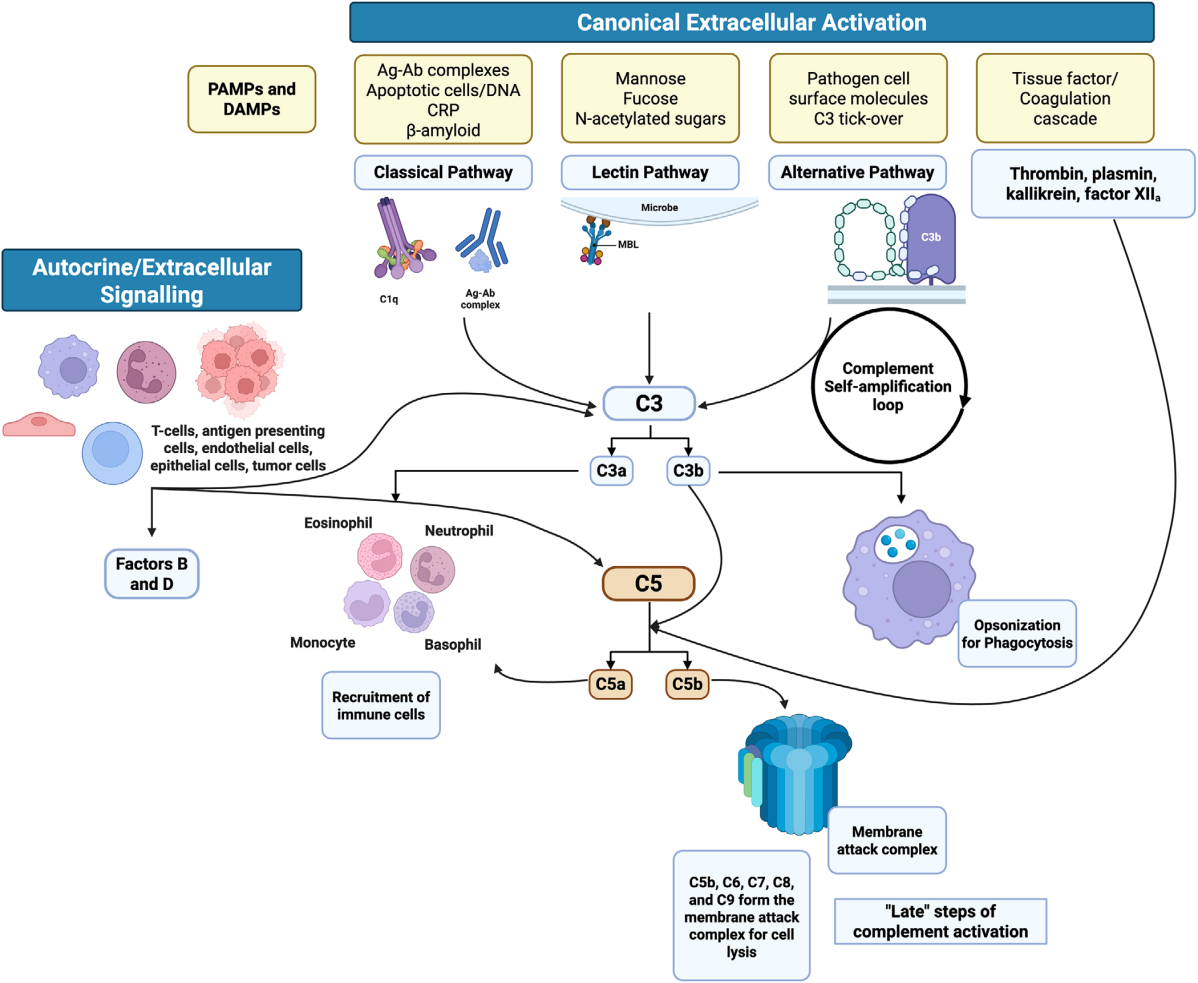
An overview of extracellular complement activation. Canonical complement activation via the classical, lectin and alternative pathways and the coagulation cascade. Autocrine complement protein secretion occurs in T cells, antigen‐presenting cells and endothelial cells. Created with BioRender.com.

All pathways converge on the central component C3, generating activation products C3a/C3b and downstream C5a/C5b, culminating in anaphylatoxin release, opsonisation and membrane attack complex (MAC) assembly. Beyond hepatic sources, extracellular complement proteins (namely, C3 and C5) can also be secreted by macrophages, monocytes, endothelial cells, epithelial cells, natural killer (NK), dendritic cells (DC), cancer cells, B and T lymphocytes^3^, ^4^ (Figure 2).

## 2 INTRACELLULAR COMPLEMENT: THE COMPLOSOME

While historical attention has focused on the complement system as an extracellular component of innate immunity, intracellular complement known as ‘the complosome’ has been recently identified as a key cell‐intrinsic signalling programme that integrates metabolism, autophagy, immune activation and gene regulation.^1^ First defined in CD4^+^ T cells in 2013, intracellular complement activation and endogenous C3 and C5 protein production were revealed as essential for T‐cell homeostasis and pro‐inflammatory cytokine production.^5^ Since then, complosome activity has been detected in monocytes, macrophages, dendritic cells, epithelial and cancer cells, among others.^1^, ^6^

### 2.1 Generation of intracellular complement effectors

Intracellular complement signalling is mediated by C3aR1 and C5aR1/C5aR2, which can signal from endosomal/lysosomal compartments and, in defined contexts, from mitochondria or the plasma membrane of immune and non‐immune cells.^7^ The transmembrane cofactor/receptor CD46 is a second hub of complosome control: C3b (and C4b) engagement of CD46 triggers intracellular cascades (via CYT‐1/CYT‐2 cytoplasmic tails) that couple nutrient sensing and transcriptional programmes to complement status.^8^ T‐cell receptor (TCR) stimulation in T cells and Toll‐like receptor (TLR) activation in antigen‐presenting cells can drive de novo C3/C5 generation, with cathepsin‐dependent cleavage proposed as one route to yield C3a/C3b/C5a intracellularly; precise enzymology remains incompletely defined.^9^ In parallel, stores of C3 and C5 have been observed within intracellular vesicles (endosomes and lysosomes) of multiple cell types, facilitating intracellular complement activation when transported out of the cell membrane or vesicle, where autocrine re‐engagement with C3aR and C5aR is possible.^5^ CD46 signalling (via CYT‐1 nuclear shuttling and metabolic cues) has been linked to upregulated C3/C5 transcription, further reinforcing the complosome loop.^10^

## 3 COMPLEMENT AND CANCER

Mounting evidence places the intracellular complement programme (complosome) at the interface of tumour biology and immunity, linking cell‐intrinsic signalling to T‐cell dysfunction, myeloid remodelling, and therapy resistance in the tumour microenvironment. Local complement activity is detected across cancers, supporting a role in the tumour microenvironment (TME) (Table 1).

**TABLE 1:** Evidence of complement protein dysregulation in cancer.

Protein	Cancer type	Description	References
**Overexpression of complement proteins in cancer**			
C1q	Cervical cancer	Increased levels of C1qB protein are found in serum and cervical cancer tumour tissue. C1qB expression positively correlated with Ki67 and P16 expression	11
	Melanoma, colon adenocarcinoma, lung adenocarcinoma, breast adenocarcinoma, pancreatic cancer	Increased levels of C1q in tumour stroma and vascular endothelium, mainly expressed by vascular endothelial cells, fibroblasts and myeloid cells	12
	Glioblastoma	Serum C1q upregulated in glioblastoma patients vs. healthy matched tissue, and C1q marked deposition within tumour tissue	13
C1s	Cutaneous squamous cell carcinoma (cSCC)	Overexpression of C1s in cSCC observed in cell lines and patient samples. Knockdown of C1s promotes apoptosis and growth suppression in vitro	14
	Renal cancer	Overexpression of C1s in tumours independently associated with high infiltration of macrophages and T cells in patient samples and associated with poor prognosis	15
	Prostate cancer	Serum C1q upregulated in prostate patients vs. healthy matched tissue	16
C1qBP	Lung cancer	Overexpression of C1qBP in patient lung cancer samples compared to healthy tissue	17
C1r	SCC	Overexpression of C1r in SCC observed in cell lines and patient samples. Knockdown of C1r promotes apoptosis and growth suppression in vitro	14
	Melanoma	Overexpression of C1r in patient samples of melanoma vs. healthy tissue	
C3	Gastric cancer	Overexpression of C3 in gastric cancer tissue compared to healthy tissues	18
	Pancreatic cancer	C3 overexpressed in PDAC tissue, and its expression correlates with metastatic potential	19, 20
C3a	cSCC	C3a overexpression in patient tumour tissues correlated with tumour cell growth in vitro	21
	Oesophageal adenocarcinoma	Overexpression of C3a in oesophageal adenocarcinoma tissue compared to healthy tissues	22
	Breast cancer	Overexpression of C3a in serum of breast cancer patients compared to healthy patients	23
C3b	NSCLC	Overexpression of C3 observed in paclitaxel resistance of NSCLC cells; knockdown of C3 promotes apoptosis	24
C4a	Papillary thyroid cancer	Overexpression of C4a in serum of patients	25
C4d	Renal cancer	Overexpression of C4d in plasma of renal cancer patients and deposits at tumour site	26
C9	Gastric cancer	C9 overexpression in the sera of gastric cancer patients vs. healthy controls	27
CD55	Colon cancer	CD55 overexpression associated with less differentiated, higher grade tumour tissue in patient samples	28
CD59	Colon cancer	CD59 overexpression associated with less differentiated, higher grade tumour tissue in patient samples	28
CD46	Bladder cancer	CD46 overexpressed in bladder cancer compared to healthy tissue, and CD46 expression inversely correlated with tumour stage, grade and disease progression risk	29
**Underexpression of complement proteins in cancer**			
C1q	Prostate cancer	C1q downregulated in benign prostatic hyperplasia and prostate cancer tissue, inactivating tumour suppressor WWOX	30
C1s	Ovarian cancer	Downregulation of C1s mRNA observed in diseased tissue vs. healthy control	31, 32
	Lung cancer	Downregulation of C1s at site of lung cancer tumour tissue compared to peritumoral tissue	17
C4BP	Ovarian cancer	Downregulation of C4BP mRNA in ovarian cancer tissue compared to healthy control	32
C7	Ovarian cancer	Downregulation of C7 mRNA in ovarian cancer tissue compared to healthy control	32
CD55	Ovarian cancer	Downregulation of CD55 in ovarian cancer tissue compared to healthy controls	33

In the clinic, among glioblastoma multiforme patients, elevated C3 and C5b levels were observed in tumour tissue versus healthy controls.^13^ Elevated deposition of C3 has also been observed in patient samples of non‐small cell lung cancer (NSCLC), gastric cancer and pancreatic cancer.^18^, ^19^, ^34^ High deposition of C3 has been significantly associated with poor overall 5‐year survival in gastric cancer patient samples, and was significantly associated with pancreatic cancer metastasis.^18^, ^19^ The effector anaphylatoxin C3a has also been shown to have increased deposition in tumour cell tissue when compared to healthy control tissue in cutaneous squamous cell carcinoma (cSCC), breast cancer and oesophageal adenocarcinoma.^35^ Additionally, elevated complement proteins are frequently found in sera from cancer patients versus healthy controls. Elevated C3a protein has been observed in the sera of breast cancer patients, and elevated C3a in breast cancer tissue is associated with lymph node metastasis.^23^, ^36^

Functionally, complement proteins produced both intracellulary and extracellularly have been shown to have a direct impact on tumour growth and therapeutic response. In a lung cancer murine model, silencing of C3 inhibited tumour growth,^37^ and in NSCLC models, nuclear C3b is enriched in paclitaxel‐resistant cells, implicating complement in drug resistance.^24^ Clinically, complement proteins have been linked to treatment response and resistance. In colorectal (CRC) cancer, high C3 expression was associated with increased FOLFOX chemotherapy resistance and poorer prognosis.^38^ Although complement classically enables cell killing, the complosome in immune and tumour cells can promote a pro‐tumour, immunosuppressive TME via myeloid recruitment, T‐cell metabolic/exhaustion programmes and altered cytokine networks.^39^ Here, we map the complosome–immune microenvironment interface in cancer, examining how these circuits may underpin treatment resistance, tumour response and inform actionable interventions.

Despite increasing evidence supporting a pro‐tumour role for intracellular complement signalling, it is important to highlight that findings across studies are not uniformly concordant. While multiple reports demonstrate that intracellular C3 and C5 activation promotes tumour cell survival, immune evasion and metabolic adaptation, other studies suggest that complement signalling may exert context‐dependent or even antitumour effects under specific conditions.^40^, ^41^ For example, discrepancies have been reported depending on tumour type, stage and immune composition, as well as whether complement activity is assessed acutely versus chronically.^42^, ^43^ These divergent observations highlight that complosome signalling is not inherently oncogenic, but instead reflects a context‐sensitive regulatory axis whose functional consequences depend on cellular state and microenvironmental cues.

## 4 COMPLEMENT, IMMUNE ACTIVATION AND T CELLS IN CANCER

Rather than functioning through isolated signalling pathways, intracellular complement activation integrates metabolic, transcriptional and inflammatory programmes that collectively shape immune cell fate and tumour behaviour. In both immune and malignant cells, the complosome acts as a central organising axis that links intracellular proteolysis of complement components to downstream metabolic rewiring, cytokine output and cell survival. Understanding how these pathways converge, rather than considering them as independent observations, is essential for appreciating the role of the complosome in immune activation, immune suppression and therapy resistance.

Immunologically, intracellular complosome and complement proteins secreted by tumour and/or immune cells play a significant role in the housekeeping of immune responses, particularly among T cells. CD4^+^ and CD8^+^ T cells are the two lymphocyte subsets that are crucial for cell‐mediated immunity and the activation of cytotoxic immune responses to tumour cells.^44^ CD4^+^ and CD8^+^ T cells contain low but measurable intracellular stores of C3/C5, with de novo synthesis upregulated following TCR or cytokine stimulation.^3^, ^45^ It has been shown that intracellular cathepsins (CTSL) can then cleave C3/C5 to generate C3a/C5a, supporting cell‐intrinsic complosome signalling.^5^, ^46^

Early infection models implicated complement in T‐cell responses; C3 was first suggested to mediate CD4^+^ and CD8^+^ T‐cell responses in 2002, where mice deficient in C3 had significantly lower lymphocyte responses in a preclinical model of influenza.^47^ However, the link between complement and T‐cell‐mediated anticancer immunity was not identified until 2008 by Markiewski et al., when TME‐derived C5a was shown to suppress antitumour CD8^+^ T‐cell responses, enhance tumour growth, increase production of reactive oxygen species (ROS), nitric oxide synthase (NOS) and immunosuppressive myeloid‐derived suppressor cells (MDSCs) in a murine cervical cancer model.^48^ Subsequent work has linked complement to dampened anticancer CD4^+^/CD8^+^ responses across models, including C5aR1‐dependent suppression and C3a‐driven restraint of CTL proliferation/apoptosis programmes.^48^, ^49^, ^50^ Vadrevu et al. demonstrated that expression of C5aR promotes metastases in a breast murine model by suppressing CD8^+^ and CD4^+^ T‐cell populations and function in lung tissue.^49^ C3a has also been shown to be specifically expressed by immune cell populations and promotes T‐cell apoptosis and inhibition of CTL proliferation.^51^

### 4.1 Potential complosome–immune axis mechanisms underlying carcinogenesis

**FIGURE 3:**
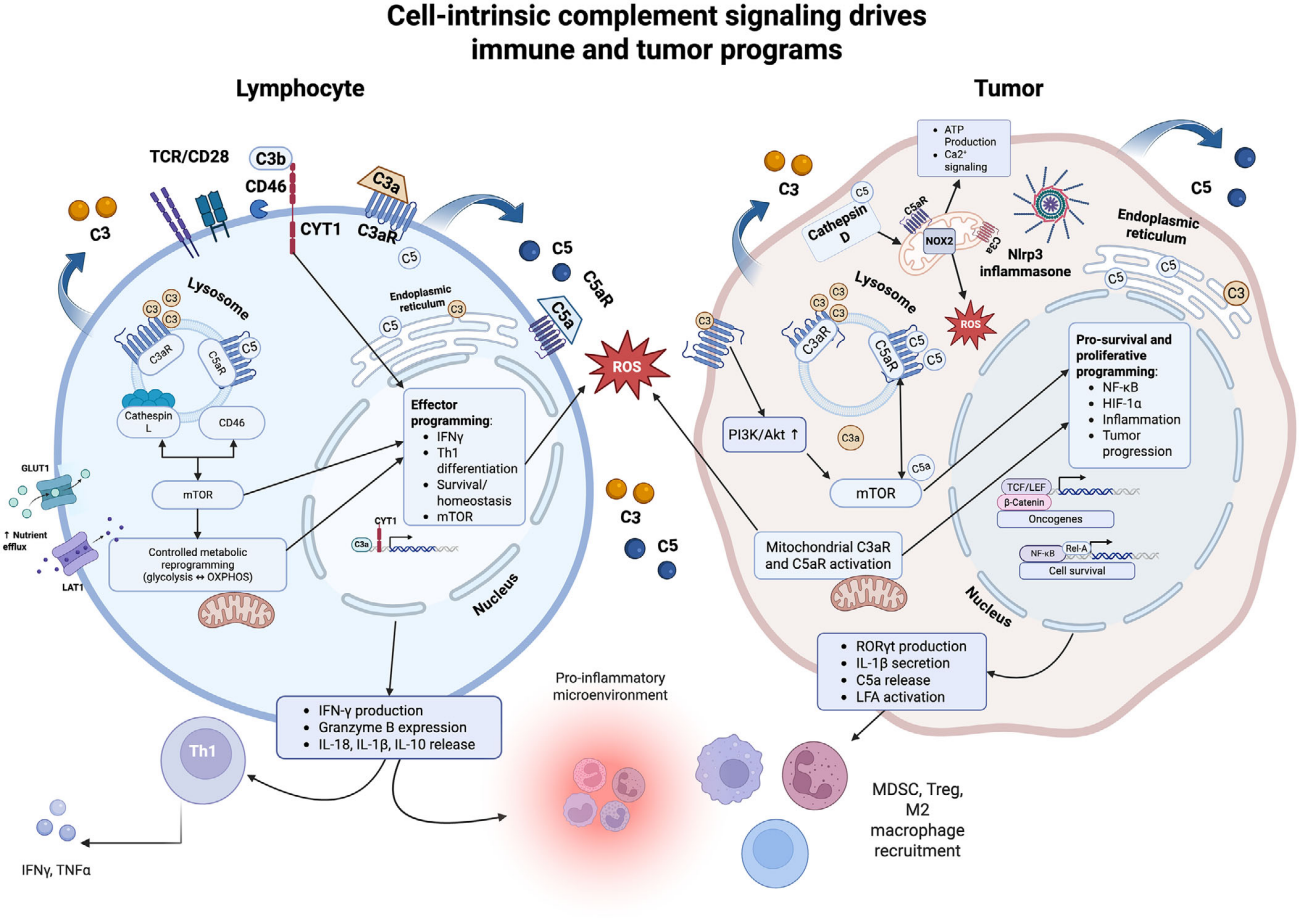
An overview of the complosome signalling dynamics governing immune activation in both immune and cancer cells. Schematic illustrating parallel but functionally divergent complosome signalling pathways in lymphocytes (left) and tumour cells (right). In lymphocytes, intracellular stores of C3 and C5 are cleaved within endolysosomal compartments, generating C3a and C5a that signal through intracellular and surface‐localised C3aR and C5aR, as well as CD46. Engagement of these receptors coordinates metabolic reprogramming, including increased glycolysis, nutrient uptake via GLUT1 and LAT1, mTOR activation, and controlled reactive oxygen species (ROS) production, supporting T‐cell survival, Th1 differentiation and effector functions such as IFN‐γ production and cytotoxic mediator release. In tumour cells, cell‐intrinsic complement activation similarly engages C3aR and C5aR localised to endosomes, lysosomes, mitochondria and the endoplasmic reticulum. Downstream intracellular complosome signalling preferentially activates pro‐survival and pro‐tumorigenic pathways, including PI3K‐AKT‐mTOR signalling, NF‐κB and HIF‐1α transcriptional programmes, inflammasome activation and sustained ROS production. These pathways promote metabolic adaptation, resistance to stress, inflammatory signalling and tumour progression, while shaping an immunosuppressive microenvironment through recruitment of myeloid‐derived suppressor cells (MDSCs), regulatory T cells and M2‐polarised macrophages. *Key unanswered questions include the temporal regulation of intracellular complement activation and the extent to which complosome signalling is conserved across tumour types and immune subsets*. Created with BioRender.com.

Defects in antitumour CD8^+^/CD4^+^ function reflect complosome‐connected mechanisms: (i) T‐helper differentiation, (ii) metabolic reprogramming, (iii) APC‐mediated suppression and (iv) CD46 signalling^52^ (Figure 3). In T cells, intracellular complosome activity is mainly mediated by C3aR1/C5aR1/C5aR2, which can signal from the plasma membrane, endosomes/lysosomes and in defined contexts, mitochondria.^5^ Upon activation of these receptors on and within T cells, increases in cellular glycolysis, oxidative phosphorylation (OXPHOS), mTOR signalling, nutrient efflux, ROS and pro‐inflammatory cytokines (context‐dependent [IL‐12, IL‐13]) have been observed.^53^ However, T cells are not the only immune subpopulation shown to mediate or be mediated by complosome activation. In a sentinel study by Liszeski et al., both basal ‘resting state’ intracellular stores of C3 and ‘tonic’ intracellular C3a have been observed in myeloid (monocytes, neutrophils), lymphoid (CD8^+^ T cells, B cells), epithelial, endothelial cells and fibroblasts.^5^ While the complosome is most well characterised in T cells, intracellular complement seems to be a ubiquitous phenomenon across immune cell populations. Additionally, complosome activity within tumour cells themselves has the potential to recruit immunosuppressive MDSCs,^54^ impair T‐cell function^55^ and polarise macrophages to a pro‐tumour phenotype.^56^

Collectively, these findings support a model in which intracellular C3 and C5 cleavage initiates a cascade of receptor‐dependent signalling events that converge on metabolic reprogramming and transcriptional control. In lymphocytes, this integration canonically supports controlled effector differentiation and subsequent resolution, which may be dysregulated in cancer contexts, whereas in tumour cells, chronic complosome activation reinforces inflammatory signalling, metabolic autonomy and immune suppression.^9^, ^57^ Framing these observations within a unified mechanistic model clarifies how individual molecular findings relate to broader immune and tumour phenotypes.

### 4.2 Complosome, homeostasis and Th differentiation

Intracellular complement activation is an important mediator of T‐cell differentiation (particularly antitumour Th1 production, a subset of CD4^+^ T cells) and maintenance, tightly regulated by the production of interferon (IFN)‐γ and intracellular C3 cleavage.^58^, ^59^

Upon TCR and CTSL (ubiquitously expressed lysosomal endopeptidase) continuously cleaves intracellular stores of C3 into effector C3a and C3b, which are either exported to the cell surface or initiate intracellular transcription of interferon IFN‐γ following Th1 differentiation.^5^ As such, C3‐deficient systems show impaired Th1 and reduced IL‐4/IL‐5/IL‐13/IFN‐γ after antigen stimulation. For example, C3‐knockout mice have been shown to secrete less IL‐4, IL‐5, IL‐13 and IFN‐γ in response to antigen stimulation, limiting potential antitumour Th1 response dependent on IFN‐γ.^60^ In people, patients deficient in C3 or CD46 (co‐stimulator of IFN‐γ Th1 cell induction) have been shown to be deficient in Th1 cells and experience frequent infections.^61^, ^62^ Inhibition of C3 in vivo has also been shown to reduce Th cells producing IL‐4 (Th2), IL‐17 (Th17), IL‐2 and TNF‐α, reduce naïve CD4^+^ cells, and reduce the proliferation of both CD4^+^ and CD8^+^ cells.^63^ Along with intracellular C3, C3aR and C5aR1 have also been shown to be required for adequate Th1 differentiation. Mice deficient in either C3aR or C5aR1 on T cells and APCs exhibited reduced Th1 expansion in multiple in vivo studies.^64^, ^65^ In a model of bacterial response to *Listeria monocytogenes* (LM), C3‐deficient mice showed blunted Ag‐specific CD4^+^/CD8^+^ expansion during infection.^66^

In the context of cancer, elevated Th1 cells have been associated with a favourable prognosis in multiple cancer types, including NSCLC, ovarian cancer, breast cancer, melanoma, glioblastoma and CRC.^67^, ^68^, ^69^, ^70^ Thus, dysfunctional Th differentiation, mediated by dysfunctional intracellular complement signalling or C3 protein deficiency, is a plausible mechanism of immunosuppression in cancer.

Aside from differentiation, intracellular complement is an important component of homeostasis. Intracellular C3a has been shown to engage with C3aR on lysosomes to activate and sustain mTOR, a potent regulator of glycolysis, inhibitor of immunosuppressive Treg differentiation and promoter of T‐cell survival and homeostasis.^5^, ^71^ When C3a is blocked, swift T‐cell apoptosis occurs.^5^ Importantly, extracellularly produced C3a from serum is unable to rescue and reinstate T‐cell proliferation in T cells deficient in intracellular C3/C3a,^5^ indicating that the intracellular C3/C3a axis is necessary to maintain T‐cell function.

### 4.3 Complosome and metabolic reprogramming of T cells

T‐cell fate and effector functions of all immune cells are coupled to cellular metabolism, and there is growing evidence that shifts in metabolic pathways by the complosome shape adaptive immune responses of CD8^+^ cells.^72^ In the inactive state, T cells preferentially rely on OXPHOS to generate ATP,^73^ whereas activated and proliferating T cells are known to upregulate glycolysis.^73^, ^74^ Chang et al. demonstrated that when glycolysis is blocked in vitro by galactose, CD4^+^ T cells exhibit minimal IFN‐γ and IL‐2 production, stunting Th1 expansion, and CD8^+^ cells show reduced proliferation, showcasing the increased glycolytic need to sustain effector function.^75^

There is growing evidence that the complosome may be implicated in this metabolic shift towards glycolysis, facilitated by intracellular stores of C3/C3a in CD4^+^ and CD8^+^ T cells (predominantly produced by lysosomes and the endoplasmic reticulum).^9^ Complosome signalling contributes to this metabolic shift, as C3aR1/C3 engagement (in T‐cell lysosomal compartments) has been shown to maintain basal mTOR (regulator of glycolysis) and GLUT1 (glucose transporter 1) expression.^46^ However, it is unknown if upregulation of C3aR or intracellular C3 drives aberrant hyperactivation of glycolysis. TCR activation results in the cleavage of stored intracellular C5 by cathepsin D, generating C5a. C5a binds to mitochondrial C5aR1 and has been shown to activate glycolytic flux in neutrophils, and it is likely this process also occurs in T cells due to similar C5aR1 expression levels; however, this has still to be demonstrated in the T‐cell population.^76^, ^77^

Stimulation of TCR and co‐stimulation of CD28 in CD4^+^ T cells shuttles intracellular C3b (produced from endogenous C3) to cell‐surface CD46.^78^ CD46 couples complement to nutrient programmes: C3b–CD46 triggers γ‐secretase cleavage and CYT‐1 nuclear translocation, inducing LAT1, GLUT1 and LAMTOR1, thereby increasing amino acid/glucose uptake and supporting Th1 differentiation and IFN‐γ.^1^ In a study by Kolev et al., activation of CYT‐1 was required for the activation of LAT1 and increased expression of GLUT1 and LAMTOR1 in human T cells.^46^ When GLUT1 and LAT1 are upregulated, nutrient efflux enhances intracellular uptake of glucose and essential amino acids to support increased glycolysis and Th1 differentiation.^79^ Unsurprisingly, T cells from patients with CD46 deficiency show dysfunctional Th1 cell induction and decreased glycolysis and OXPHOS.^80^

Maintenance of T‐cell homeostasis by the complosome introduces a delicate balance of pro‐ and antitumorigenic signals. While Th1 expansion is associated with antitumour responses, the upregulation of GLUT1 and LAMTOR1 is associated with the induction of an immunosuppressive microenvironment and hyperproliferation in both immune and tumour cells.^81^

### 4.4 C3aR/C5aR axis and immunosuppression

C3aR and C5aR are highly expressed on tumour and immune cells in preclinical and patient samples.^82^, ^83^, ^84^ Interestingly, while preclinical studies suggest C3aR and C5aR are significantly upregulated in murine and patient tumours and tumour‐infiltrating lymphocytes (TILs), peripheral CD8^+^ T cells from non‐tumour‐bearing mice exhibit a lack of expression of both receptors.^85^ Activation of C5aR intracellularly by C5 cleavage by cathepsin D (CTSD) has been shown to promote carcinogenesis in CRC, via the formation of a C5a/C5aR1/KCTD5/cullin3/Roc‐1 complex, which stabilises β‐catenin and drives downstream oncogenic signalling.^76^ The increased transcription of stabilised β‐catenin results in enhanced transcription of target oncogenes Cox2, cyclin D1 and c‐Myc.^76^ Intrinsic WNT/β‐catenin signalling in tumour cells is also associated with poor T‐cell infiltration, and WNT/β‐catenin signalling in T cells has been shown to promote the expression of RORγt, a signature of Th17 cells (potent activators of Tregs).^86^, ^87^

Inflammasome crosstalk adds complexity to the immunosuppressive function of the complosome. The C5aR signalling axis is required for the assembly of the intracellular protein ‘inflammasome’ complex, which promotes maturation of pro‐inflammatory IL‐18, IL‐1β, release of pro‐inflammatory HMGB1 protein and caspase‐1‐dependent pyroptosis.^88^ Although the inflammasome is canonically activated by extracellular PAMP recognition, the inflammasome is also activated via C5a binding intracellularly to the mitochondria, with mitochondrial C5aR2–ROS–NOX2 implicated in activation.^89^

Inflammasomes play a nuanced role in cancer, and are associated with promoting angiogenesis and immunosuppression in some cancers, such as breast,^90^ while supporting Th1 and NK cell activity in others, such as CRC.^91^ In a murine C5aR2 knockdown model, C5aR2 deficiency inhibited NLRP3 activation in vivo, indicating the importance of intracellular complement signalling in inflammasome formation.^83^

### 4.5 CD46 signalling

CD46, expressed on all nucleated cells, integrates complosome with T‐cell fate and antitumour response. In its inactivated state, CD46 maintains T‐cell homeostasis by binding to Jagged‐1, shunting Notch signalling and T‐cell differentiation.^61^ When T cells are activated by the TCR, CD46 is shed, and Notch is induced, triggering Th1 differentiation.^61^, ^92^ CD46 also governs resolution of Th1 responses, driving IL‐10 and T‐cell plasticity. When CD46 is activated, IL‐10 is expressed in CD4^+^ cells and Th1 cells, inducing a switch in cytokine production from IFN‐γ to IL‐10.^93^ Clinically, CD46 is generally overexpressed in cancers, including ovarian, hepatocellular and breast cancers.^94^ Given its role in IL‐10 induction, dysregulated CD46–IL‐2R/Notch axes may contribute to tumour immune evasion.

## 5 COMPLOSOME AND IMMUNE CELL DYNAMICS

### 5.1 Regulatory T cells

**FIGURE 4:**
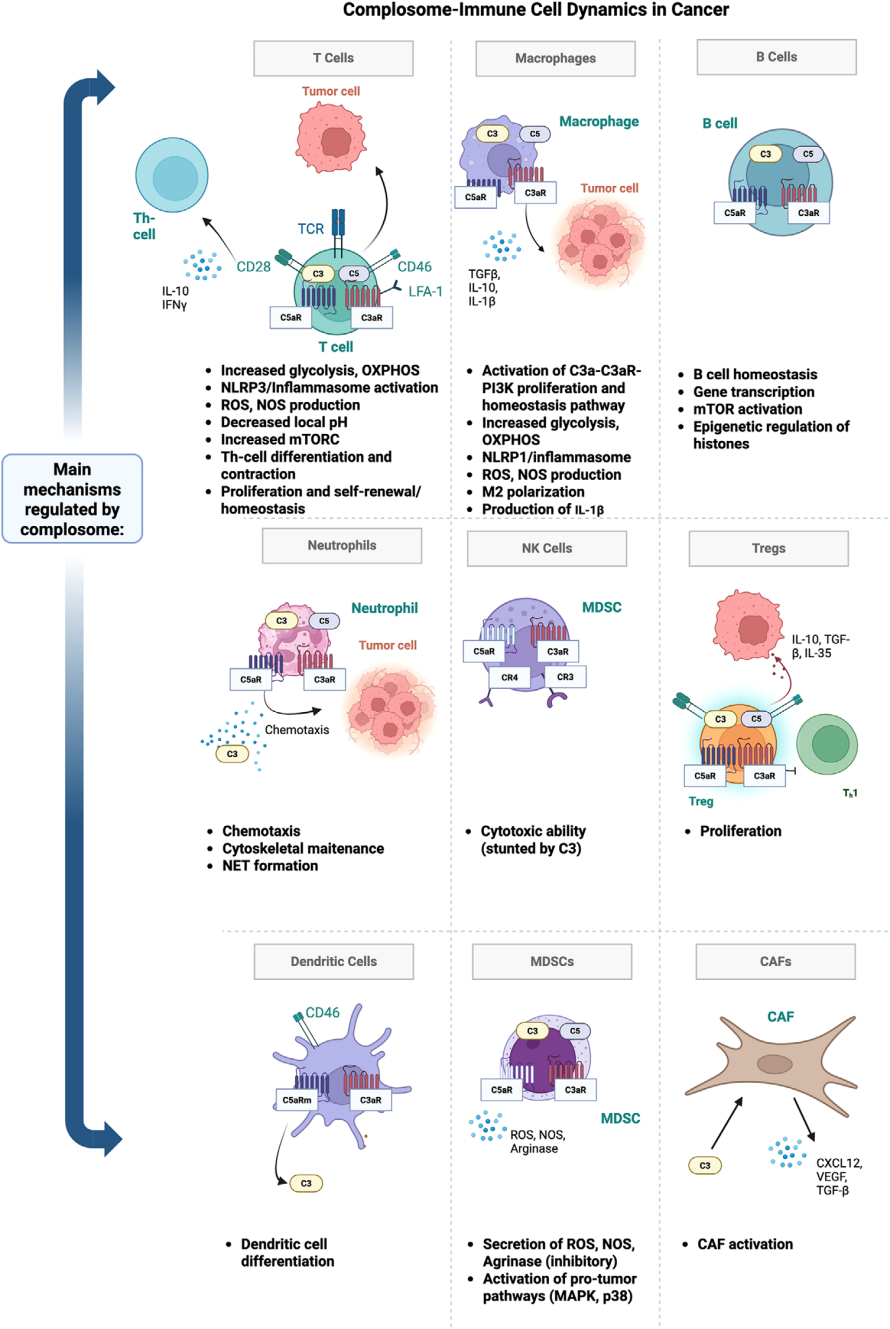
Overview of immune mechanisms regulated by intracellular complement proteins. In T cells, TAMs and Tregs, complosome signalling drives metabolic reprogramming, inflammation and immune suppression. Neutrophils and MDSCs are activated to support chemotaxis and pro‐tumour signalling, while NK cells’ cytotoxic function is inhibited. Additionally, CAFs are primed by intracellular C3 to secrete factors including CXCL12 and TGFβ, enhancing immune evasion by shunting T‐cell expansion and promoting tumour growth.

Regulatory T cells (Tregs) are thought to be in part regulated by the complosome and complement proteins, and play important roles in maintaining self‐tolerance (Figure 4). However, in cancer, Tregs promote tumorigenesis via the production of suppressive cytokines (TGFβ and IL‐10) and stimulation of angiogenesis via the secretion of VEGF.^95^ Many tumour tissues are enriched in immunosuppressive Tregs, and having a higher proportion of Tregs is associated with worse overall survival and risk of recurrence in multiple cancers, including CRC, NSCLC and triple‐negative breast cancer (TNBC).^96^, ^97^, ^98^, ^99^, ^100^, ^101^, ^102^

Higher numbers of FOXP3^+^ Tregs were observed in C3‐deficient mice, suggesting C3 may contribute to blocking Treg differentiation.^103^ Elevated Treg populations have also been observed in systemic lupus erythematosus (SLE) patients with lower plasma levels of C3 and C4.^104^ Interestingly, complement C4 protein has been shown to induce Treg differentiation directly in an in vitro model, where C4 was co‐cultured with T cells and DCs isolated from patients with SLE.^105^ While C3 and C4 are likely linked to Treg expansion, the mechanisms regulating this association remain largely unknown.

### 5.2 B cells

B cells can be pro‐ or antitumour depending on the microenvironmental context. High B cell infiltration is often associated with longer survival in breast, ovarian, renal and lung cancers, in part through the formation of tertiary lymphoid structures (TLS) with DCs and T cells that enhance antigen presentation and T‐cell priming.^106^, ^107^ TLS are lymphoid aggregates that form de novo in non‐lymphoid tissues in response to chronic inflammation or cancer, and are commonly found within and around cancer tissue.^108^ Conversely, B regulatory cells (Bregs)—driven by cytokines, such as IL‐21, IL‐6, IL‐33, IL‐35, IL‐1β and IFN‐α, secrete IL‐10 and promote immunosuppression.^109^, ^110^, ^111^

Within this spectrum, the complosome is a core regulator of B‐cell physiology. B cells contain intracellular C3/C3a and can generate C3 autocrinally, which is necessary for B cell self‐renewal and survival; serum‐derived C3 can also be imported and processed via cathepsin L to C3b/C3a, with nuclear entry reported for exogenous C3.^9^, ^10^, ^112^ B cells express C3aR1/C5aR1, and genetic disruption of these receptors abrogates mTOR activation after BCR/CD40 stimulation, leading to premature germinal‐centre collapse and defective maturation—highlighting a requirement for complosome signalling in B‐cell activation.^113^, ^114^

Complosome signalling in B cells has also been associated with epigenetic modulation of gene expression. C3a inhibits histone H1 binding to DNA in a dose‐dependent manner.^10^ In cancer, the knockdown of H1 in breast cancer cells has been associated with altered gene expression, type I IFN production and a hyperproliferative phenotype.^115^

### 5.3 DCs

DCs orchestrate antitumour T cell responses through antigen presentation and co‐stimulation, and they express C3aR along with complement regulators CD49, CD55 and CD59.^116^, ^117^ Intracellular deficiency of C3 has been associated with impaired DC differentiation; however, the mechanism by which this occurs remains poorly understood.^118^ DCs have produced and synthesised C3 intracellularly in vivo and in vitro, and C3‐inhibited DCs exhibit reduced ability to stimulate alloreactive T cells, polarise CD4^+^ T cells to a Th2 phenotype and drive Treg expansion.^119^

Beyond differentiation, the complosome in DCs modulates antigen handling and the tempo of T cell priming. Whereas extracellular opsonisation tags targets via C3b for phagocytosis, DCs themselves display C3 fragments, enabling a form of intracellular opsonisation that shapes antigen processing.^120^ In apoptotic cell models, C3‐sufficient DCs retained intact cargo (lysosomal debris) longer, indicating that C3 fragments act as chaperones of phagosome maturation. Conversely, C3‐deficient DCs digested cargo rapidly and elicited diminished CD4^+^ proliferation, effectively pacing down the T cell response.^121^ Unsurprisingly, T cell response was ‘paced’, as DCs deficient in C3 consumed the target antigen faster and elicited diminished CD4^+^ T cell proliferation compared to the C3‐sufficient DCs, which had not digested the apoptotic cell by endpoint and elicited a more robust T cell response.^121^ These data support a role for intracellular C3 in regulating antigen‐trafficking kinetics and MHC‐II exposure, with potential implications for cancer: reduced DC C3 could truncate antigen presentation and blunt antitumour immunity.

### 5.4 Neutrophils

As the most abundant innate immune cells in bone marrow and blood, neutrophils have also been associated with promoting cancer progression and poor prognosis when polarised to a tumour‐associated ‘TAN’ state.^122^, ^123^ TANs inhibit CD8^+^ T cells and secrete MMP9, VEGF and Arg1.^124^ Higher neutrophil infiltration in tumour stroma is associated with poorer prognosis in a variety of cancers, including breast, lung and glioma.^125^, ^126^ The link between complement and neutrophils was first identified in 1993, when C3 was characterised as a strong chemoattractant for neutrophils in vivo.^127^ The role of C3 in neutrophil chemotaxis was validated more recently in a murine model of acute pancreatitis, where C3 expression was required for the recruitment of neutrophils to the pancreas.^128^ As an innate immune cell, neutrophils are known to have potent intracellular stores of C3, and intracellular complement likely mediates neutrophil cytoskeletal maintenance.^129^ In a study assessing neutrophils from healthy controls and SLE patients, neutrophils from healthy patients exhibited upregulated C3 and were associated with the preservation of cytoskeletal organisation.^130^ In the context of SLE, cytoskeletal abnormalities impair TCR signalling and immune synapse formation between T cells and APCs, which contribute to disease progression.^131^ However, in cancer, increased C3 may also promote a pro‐tumour TAN phenotype. In a C3‐knockout murine model of ischaemia‐reperfusion injury, C3 was a requirement for increased neutrophil recruitment to the injury site and neutrophil extracellular trap (NET) formation.^132^ NETs are composed of condensed nuclear and mitochondrial DNA, proteases and pro‐inflammatory mediators, which may promote EMT and/or activate dormant cancer cells.^133^

### 5.5 Natural killer cells

Natural killer (NK) cells limit tumour growth via granzyme/perforin‐mediated cell killing and the production of inflammatory cytokines and chemokines. Like CD8^+^ cells, NK cells can become dysfunctional in the TME as a result of stromal interactions with immunosuppressive cancer‐associated fibroblasts (CAFs), tumour‐associated macrophages (TAMs) and MDSCs, preventing cancer cell killing.^134^

NK cells highly express C3aR, C5aR, C5aR2, CR3 and CR4; however, it is unknown whether NK cells can secrete or produce C5 and/or C3 intracellularly.^135^ Interestingly, in a C3‐knockout murine melanoma model, C3 deficiency resulted in increased antitumour immunity of NK cells and reduced tumour growth.^136^ Depletion of C3 has also been shown to enhance the ability of target cells coated with rituximab to activate NK cells and improve the efficacy of monoclonal antibody therapy in a murine lymphoma model.^137^ Similarly, C3aR signalling has been shown to inhibit NK infiltration in vivo by triggering the formation of high‐affinity LFA‐1, a critical lymphocyte trafficking integrin, suggesting that C3/C3aR blockade may support anticancer NK responses.^138^

### 5.6 Macrophages

Macrophages are early, prevalent TME infiltrates. When polarised to M2‐like (pro‐tumour) TAMs, they secrete immunosuppressive cytokines and ROS, and higher TAM burden predicts poorer outcomes in pancreatic, gastric and breast cancers.^139^ Higher infiltration of TAMs has been associated with poorer prognosis in numerous cancers, including pancreatic, gastric and breast cancer.^140^, ^141^, ^142^

Intracellular C3 in tumour cells is associated with the promotion of an immunosuppressive macrophage phenotype. Zha et al. demonstrated that intracellular activation of tumour‐derived C3 (resulting in the intracellular generation of C3a and C3b) promoted the generation of TAMs via activation of the C3a–C3aR–PI3K pathway in vivo.^143^ Importantly, the activation of PI3K has been shown to activate other pro‐tumorigenic pathways in TAMs, such as NF‐κB, which functions to sustain hyperproliferation and immunosuppression.^144^ This generation of M2‐like macrophages was also associated with the suppression of CD8^+^ T cells, a common interaction between TAMs and T cells. Further, when C3 was deleted in high C3‐expressing tumour‐bearing mice (implanted with 4T1 mammary carcinoma cells), mice were sensitised to anti‐PD1 therapy, and reduced tumour burden was observed.^143^ The induction of C3 gene transcription can also be activated by the binding of LFA‐1, and LFA‐1 activation may inhibit NK cells and promote M2 macrophages simultaneously.^145^

Additionally, as previously described in T cells, macrophages and monocytes use autonomous, constitutive and intracellular C3 and C5 activation (engaging with mitochondrial C5aR) to sustain metabolic reprogramming and inflammasome formation.^1^, ^77^ As demonstrated by Niyonzima et al., continuously synthesised intracellular C5 shifts ATP production towards ROS generation, aerobic glycolysis and promotes IL‐1β expression in macrophages.^77^, ^146^

### 5.7 MDSCs

MDSCs are another prevalent immune cell population in the TME, implicated in immunosuppression and carcinogenesis. Like Tregs, they restrain CD8^+^ T‐cell expansion via IL‐10, arginase and ROS, and express high levels of C5aR1.^49^, ^147^ C5a, generated intracellularly and/or locally within tumours, acts as a potent chemoattractant; once released, it recruits MDSCs to tumour and stromal sites and directly signals via C5aR1 on MDSCs to enhance suppressive function.^49^, ^148^, ^149^ Consistent with local production, C5/C5a is detected within the TME and is secreted by phagocytes.^48^, ^150^ In cervical cancer models, C5aR1 sufficiency was required for the intratumoral accumulation of polymorphonuclear MDSCs.^48^ In colon cancer, anti‐PD‐1/PD‐L1 therapy increased C5a intratumorally, which amplified MDSC inhibitory activity and boosted ROS/NOS/arginase output; C5a/C5aR1 blockade restored antitumour effects of checkpoint therapy.^151^ Lastly, in hepatocellular carcinoma cells, C3 secretion induced by *PIWIL1* gene activation (upregulated in 80.4% of colon cancer cases), activated oncogenic p38 and MAPK signalling in MDSCs.^46^, ^152^

### 5.8 Fibroblasts and CAFs

Cancer‐associated fibroblasts (CAFs) are abundant stromal cells in the TME. Recent work shows that intracellular C3 can be cleaved to generate C3a/C3b, which then signals autocrinally via C3aR1 (endosomal or plasma‐membrane) to drive ‘tissue priming’ and metabolic reprogramming.^51^, ^153^, ^154^ Primed fibroblasts increase pro‐inflammatory IL‐6/IL‐8 and CCL2/CXCL12, switch to aerobic glycolysis, and encode transcriptional/epigenetic memory of past inflammatory events, heightening responsiveness to later stimuli, a phenotype commonly observed in autoimmunity and chronic inflammation, and is increasingly implicated in tumour‐associated stromal activation.^155^, ^156^ In synovial models, tissue priming depends on intracellular C3/C3a, drives glycolysis, and activates mTOR and HIF‐1α.^154^, ^157^ In the context of cancer, intracellularly generated and secreted C3a can promote cancer invasion and metastasis via the activation of CAFs in murine models.^158^

Concordant data in gastric cancer models show CAF‐intrinsic C3 associates with NF‐κB upregulation, CD8^+^ dysfunction and therapeutic resistance. Patient samples also revealed an association between increased C3^+^ CAFs in the stroma and stromal infiltration of immunosuppressive M2 macrophages.^159^ Targeting the NF‐κB/C3 pathway by silencing p65 through siRNA significantly decreased C3 secretion in CAFs; however, the exact intracellular cues that drive initial C3 cleavage are currently unknown.^159^ Taken together, CAF complosome activation, via intracellular C3 cleavage and autocrine C3aR1/CD46 signalling, drives metabolic rewiring, myeloid recruitment and broad immunosuppression within the TME.^51^, ^154^, ^156^

## 6 THERAPEUTIC TARGETING OF THE COMPLOSOME‐IMMUNE AXIS IN CANCER

Given its roles in tumorigenesis and immune regulation, the complosome is an attractive therapeutic axis, though no complement‐directed drugs are yet approved in oncology.^160^, ^161^ While anticancer complement‐inhibitory drugs are in development, most aim to target extracellular proteins, leaving the intracellular arm relatively unexplored.^162^

One leading preclinical strategy is C3aR/C5aR blockade combined with traditional immune checkpoint inhibitors. In lung cancer models, C5a–C5aR1 antagonism plus anti‐PD‐1 reduced tumour growth, prolonged survival, decreased MDSCs and restored CD8^+^ effector function.^82^, ^163^, ^164^, ^165^ In melanoma, co‐delivery of C3aR1/C5aR1 antagonists with anti‐PD‐1 produced synergy beyond PD‐1 alone, replicated across studies.^82^, ^151^, ^165^ It has also been demonstrated that C5aR blockade may reprogramme M2 TAMs and synergise with anti‐PD‐1 therapy.^166^ In a gastric cancer model, higher intratumoral densities of C5aR1^+^ TAMs, the compartment with the strongest C5aR1 signal, associate with worse overall survival and chemoresistance.^167^ Selective C5aR1 antagonism reduced tumour proliferation, lowered IL‐6/IL‐10/TGF‐β from TAMs (consistent with M1 repolarisation), and reinvigorated CD8^+^ cytotoxicity in vivo.^166^

Complement inhibition can also potentiate cytotoxic therapies. Radiotherapy increases intratumoral C3/C3b and C3a/C5a in ovarian tumour models^168^; In CRC, systemic C5aR1 antagonism improved radiation responses.^169^ Systemic blockade of C5aR1 has also shown preclinical synergistic effects with chemotherapy. C5aR1 blockade plus paclitaxel enhanced CD8^+^ proliferation/cytotoxicity in an IFN‐γ‐dependent manner in SCC.^50^ Data from our group link complement to neoadjuvant chemoradiation (nCRT) response across gastrointestinal cancers.^170^, ^171^ In oesophageal adenocarcinoma (OAC), it was demonstrated that pretreatment serum levels of complement anaphylatoxins (C3a/C4a) predict poor response to nCRT and adverse outcomes.^170^ More recently, in rectal cancer, we reported that complement is increased in radioresistant rectal cancer cells and that C3 functionally modulates the radioresponse in vitro, with inhibition of C3 reversing a radioresistant phenotype, concomitant with increased radiation‐induced DNA damage and a shift towards a more radiosensitive cell cycle phenotype.^172^ Collectively, these findings implicate complement, particularly C3 and its downstream effectors, as a determinant of resistance to cytotoxic therapy and a rational target for radio‐chemo‐immunotherapy combinations.

Therapeutically, translating these preclinical studies into the clinic largely depends on agent selection and receptor selectivity, access to intracellular complement pools and delivery to relevant cell types. Current tools, including C5aR1 antagonists (PMX53, PMX205, avacopan/CCX168) and C3aR1 tool/antagonist compounds (SB290157, context‐dependent pharmacology), primarily engage cell‐surface receptors.^165^, ^173^, ^174^, ^175^ The dual small‐molecule DF2593A blocks C3aR1/C5aR1 in vitro, and JPE1375 shows activity against C5aR1, including mitochondrial pools in select settings.^77^, ^176^ However, selective access to intracellular C3 versus extracellular C3 is limited.^1^

Despite its therapeutic promise, targeting intracellular complosome signalling presents substantial translational challenges. Key barriers include the difficulty of achieving subcellular delivery to intracellular receptors such as C5aR1,^177^ tumour microenvironment heterogeneity that may drive variable therapeutic responses, and the risk of unintended immune perturbation due to the pleiotropic roles of complement in host defence.^178^, ^179^ Additionally, most available complement inhibitors were developed to neutralise extracellular pathways and may not effectively engage intracellular targets. Emerging strategies to overcome these challenges include tumour microenvironment‐responsive nanocarriers,^180^ cell‐type‐restricted delivery platforms,^181^ and biased receptor modulators designed to selectively inhibit pathogenic intracellular signalling while preserving homeostatic immune functions.^182^ Advances in single‐cell profiling and spatial transcriptomics may further enable rational patient stratification and guide precision targeting of complosome‐driven tumour states.

Beyond therapy, the complosome shows promise as a biomarker. Profiling a patient's intracellular complosome and ‘complotype’ (complement‐gene polymorphisms) may inform prognosis and treatment selection.^172^, ^183^ Potential approaches include quantification of intracellular C3 or C5 expression, assessment of C3a/C5a signalling signatures, or integration of complotype variants with transcriptional and metabolic profiling.^36^, ^43^ However, standardised detection methods, clinically actionable thresholds, and prospective validation in patient cohorts remain largely unexplored, underscoring the need for translational studies to define the clinical utility of complosome‐based biomarkers.^184^, ^185^ While systemic complement and complosome testing are not yet routinely included in oncology patient profiling, given the role of the complosome in cancer and immune regulation, complosome proteins may be important novel biomarkers in prognostic, diagnostic and clinical decision‐making. Prospective evaluation within oncology workflows, especially among cancer types where early diagnosis is challenging, such as OAC, is warranted.

## 7 CONCLUSION

The complosome represents an emergent axis of tumour‐immune regulation with relevance to cancer progression, treatment resistance and inflammatory comorbidity. In this review, we highlight evidence across preclinical models indicating that intracellular complement shapes T‐cell expansion and differentiation, promotes immunosuppression through MDSC, macrophage and CAF recruitment/polarisation, and reprogrammes metabolism via mTOR activation, glycolytic flux and ROS/NOS. Therapeutic strategies to modulate complosome C3 activity, particularly through C3aR and C5aR antagonism, hold promise in reducing immunosuppression and pro‐tumour metabolic reprogramming in immune cells. Further research is required to unravel the exact mechanisms underlying intracellular C3 cleavage and complosome activation in tumour cells and immune cells. Future research priorities include (i) achieving compartmental specificity (selective access to intracellular vs. extracellular complement); (ii) building pharmacodynamic endpoints (myeloid trafficking; T‐cell metabolism/exhaustion) into study and trial design; and (iii) standardising complement/complosome biomarker panels for routine oncology workflows.

## AUTHOR CONTRIBUTIONS

A.B., J.L. and N.L.L. conceptualised the review. A.B. wrote the original draft manuscript and prepared the figures. All authors read and edited the final version of the manuscript.

## CONFLICT OF INTEREST STATEMENT

The authors declare that they have no conflicts of interest.

## ETHICS STATEMENT

The authors have nothing to report.
